# Supplementary material for: Single-dose testosterone administration increases men’s preference for status goods
Source: Nat Commun. 2018 Jul 3;9:2433. doi: 10.1038/s41467-018-04923-0 (PMC6030157; doi:10.1038/s41467-018-04923-0)
Supplement: Supplementary file 1 — Supplementary Information [file 41467_2018_4923_MOESM1_ESM.pdf]

Single-Dose Testosterone Administration Increases Men's Preference for Status Goods

Nave et al.

Supplementary Information:

Supplementary Methods

Supplementary Figures

Supplementary Tables

Supplementary References

Supplementary Methods:

1. Prescreening criteria

Prescreening criteria excluded everyone with relevant medical and psychological conditions (5α-reductase deficiency, Klinefelter's syndrome, brain tumor, cancer, psychiatric diagnosis/diagnoses, high blood pressure, liver disease, kidney disease, angina, cancer, hepatitis, renal/kidney impairment, history of epileptic seizures and hypersensitivity to soy/alcohol), participants using prescription drugs that could have interfered with the study (oxyphenbutazone, insulin, corticosteroids, opioids), participants who self-reported consuming illegal drugs or excessive alcohol in the previous 24 hours, and non-native English speakers.

The institutional review boards of Caltech and Claremont Graduate University approved the study, all participants gave informed consent and no adverse events occurred.

2. Tests of randomization

Age, handedness, sexual orientation, relationship and family status, personal income level, and treatment expectancy characteristics of the two groups are summarized in Supplementary Table 1. Five participants did not report their age and were therefore excluded from all analyses that include age as control. We conducted two sample t-tests for differences between T and P group characteristics to check whether random assignment resulted in balance on all such variables. None of these variables showed significant differences between the groups at a level of $p = 0.05$ (see Supplementary Table 1). Two participants (one from each group) self-reported taking T treatment on a regular basis; all analyses include all of the participants for which the relevant data has been collected, and are robust when the latter two are excluded. To reduce the potential effect of a female experimenter's presence on T-related behaviors,[1] male researchers conducted all experimental sessions.

3. Hormonal assay procedure (technical details)

Steroids were measured by LC-MS/MS using an AB Sciex Triple Quad 5500. Internal standards were added to 1 ml of saliva and the steroids extracted by C18 column chromatography with 0.1 M NH4OH wash followed by 10% acetone. Steroids were eluted from the SPE with 10% methanol in acetone and dried under nitrogen. The dried samples were subjected to derivatization—the process of transforming a compound into a derivative product of similar chemical structure—with pyridine-3-sulfonyl chloride for the estrogens (estrone (E1), estradiol (E2), and estriol (E3)). 40 µL sodium bicarbonate (50mM, pH 10) and 40 µL pyridine-3-sulfonyl chloride (3 mg/mL in acetonitrile) were added to the dried samples and incubated at 60°C for 10 minutes. After derivatization, the samples were diluted with 80 µL of water and injected for LC-MS/MS analysis, with analytical separation performed on an Agilent Poroshell 120 EC-C8 column and ionization by atmospheric pressure chemical ionization (APCI) in the positive ionization mode. Supplementary Table 2 lists each analyte along with its validation results for the lower limit of quantitation (LLOQ is jargon for the lowest level of detection with coefficients of variation (CVs) < 20% over the linear range), linear range, and the inter-assay precision from the highest concentration to the LLOQ within the linear range. When salivary hormone levels of

participants were below their LLOQ, we assigned values halfway between zero and their respective LLOQ (note that the true quantities of the hormone in the sample are never zero, even when they do not reach the detection threshold).

4. Hormonal changes following treatment and manipulation check

As expected, we found significant post-treatment differences between groups with respect to all hormones influenced by T treatment (see Fig. 1, Supplementary Table 3), either as an upstream (androstenedione) or downstream (5-alpha DHT) metabolite of T.[2] Additionally, we found a decrease in progesterone 17OH resulting from an increase in T. The changes in saliva T measures were similar in magnitude to those reported in previous studies following topical gel administration of T and progesterone.[3,4] We observed no significant differences between treatment groups in hormones that were not expected to change following short-term T treatment (e.g., aldosterone, cortisol, cortisone, melatonin) in all four saliva measurements throughout the experiment (i.e., the pre-treatment and the three post-treatment measurements). The pre-treatment and first post-treatment mean hormonal saliva levels are summarized in Supplementary Table 3; note that differences between morning and afternoon hormonal levels (of both groups) were affected by diurnal cycles.[5–7]

From assays conducted during the first 13 (out of 17) sessions of the study, we noted that 72 out of 184 pre-treatment baseline saliva samples (in both treatment groups) presented measurements with higher T levels than are expected in normal young men (greater than 400 pg/mL). All other measurements (including T metabolites) were hormonally typical. We traced the cause of these abnormal measurements to T gel transfer to common surfaces (e.g., doorknobs, mouse pads). Crucially, the high measurements were caused by local spread of T into saliva tubes, but *physiological* levels were unaffected by superficial contact with the dry nuisance T gel, as (a) we observed normal pre-treatment levels of T metabolites, namely DHT and androstenedione, in all participants; (b) none of the placebo group participants showed abnormally high values of T metabolites in any of the post-treatment measurements; (c) only five out of 118 participants from the placebo group showed consistently elevated T measurements in all three of the post-treatment saliva samples; and (d) previous investigations found that interpersonal T transfer is highly unlikely even with skin-to-skin contact.[8] Thus, we found convergent evidence that *biofluid* levels were unaffected by superficial contact.

In response to this finding during the course of the experimental period, we identified all surfaces and objects through which T could spread in the facility and improved sterile isolation protocol to eliminate the spread of the dried T gel. This protocol included thorough cleaning of keyboards, computer mice, chair backs, displays, and all doorknobs with a bleach-alcohol solution after each session as well as asking participants to carefully wipe hands with a wet tissue before collecting each saliva sample. New pens were used for each session, and all previously used pens were removed from the testing area. Clipboards and other miscellaneous objects that participants did or could interact with were cleaned, and an aerosol air sanitizer that bonds to volatile organic compounds (VOCs) was sprayed into the air. Following the adoption of this strict sterilization protocol, we found a drastic reduction in incidence of high T samples in the pre-treatment measurements, to a total of five participants out of 58 in the following four sessions (sessions 14–17).

5. Digit ratio measurements

The ratio of second (index) finger length to fourth (ring) finger (abbreviated 2D:4D) is considered a proxy for prenatal T exposure,[9] although this hypothesis is still under debate.[10,11] Participants' 2D:4D ratios were measured by two independent raters using hand scans and digital calipers (correlation between the two raters was ~0.95). The right-hand digit ratio was not calculated for one participant due to a broken finger, and therefore he was excluded from all analyses that use the right-hand digit ratio as control. Correlation between the digit ratios of the left and right hands was 0.64, p = 0.0001. There were slight differences between the two treatment groups in the right-hand 2D:4D (T: M = 0.943, SD = 0.033; P: M = 0.953, SD = 0.033; $t(240) = 2.19$, $p = 0.03$, 95% CI [–0.018 –0.001]) and no differences in the left-hand 2D:4D (T: M = 0.945, SD = 0.033; P: M = 0.947, SD = 0.033; $t(241) = –0.57$, $p = 0.56$, 95% CI [–0.011 0.006]). Regression models are reported for the right hand. All of the results hold when replacing the right-hand 2D:4D by either the left-hand digit ratio or the averaged digit ratio of both hands.

6. Mood questionnaire

To test whether our results could be due to changes in the mood state of our participants, we measured mood using the PANAS-X scale,[12] both pre-treatment (in the morning) and post-treatment (in the afternoon). Supplementary Table 4 shows a modest decrease in both affect measures over time (morning vs. afternoon) and no treatment or time x treatment interaction, indicated by the output of two-way analysis of variance (ANOVA) with an interaction term, ruling out this indirect way in which T might affect cognition and behavior. Three participants did not answer all of the negative affect items, and five participants did not complete all of the positive affect items; these participants were excluded from analyses that include these scales as control variables.

7. Extended data analysis and results for task 1

The influence of T administration on preferences for brands varying in social rank association. We estimated a linear regression mixed models with random intercepts for brand pair and participant[13] (using fixed-effect models yielded similar results). The dependent variable (DV) in all models was the preference rating (z-scored at the question level), such that higher rating implied greater preference for the brand high in social rank. All analyses were conducted using the function *lmer* implemented in R.[14]

Model A1 included only treatment (T = 1, placebo = 0) as an independent variable (IV). Model A2 also included age, positive and negative affect, treatment expectancy, and the right hand 2D:4D (results hold when the left hand or the average of the two hands is used). Model A3 included all of the IVs of model A2 with the addition of all of the hormonal levels that were not affected by the treatment, as measured from the first post-treatment saliva sample (i.e., the second overall sample). We replicated model A3's results where the measurements were replaced by either the second post-treatment saliva sample (i.e., the third overall measurement, model A4) or the baseline (morning) saliva sample (model A5). The results are summarized in Supplementary Table 7.

Testing for a dispositional effect of T on preferences for brands varying in social rank association. We repeated the analyses above (testing for the influence of T administration on task 1), this time when including either the logged baseline (pre-treatment) T levels, or the logged baseline levels of androstenedione. The latter is a downstream metabolite of testosterone, which levels correlate with testosterone (in our sample the correlation was 0.44), and that was suggested (by animal studies) to have potent behavioral effect that are similar to testosterone.[15,16]

Model F1, F2, and F3 are identical to models A1, A2, and A3, but also include the logged baseline T levels. Models F4, F5, and F6 repeated these analyses, this time with baseline levels of androstenedione instead of testosterone. The results are summarized in Supplementary Table 12.

8. Extended data analysis and results for task 2

T's influence on attitudes toward goods promoting different strategies to enhance social rank. Our main hypothesis was that T increased attitudes toward goods described as status-enhancing, as opposed to power-enhancing or higher in quality. We tested this hypothesis by estimating linear regression mixed models[13] with participant random intercepts and slopes for "power" and "status" positioning, and product random intercepts. The DV of all models was the goods' attitude score, such that a high score reflected a more positive attitude toward the good. The IVs were a binary treatment indicator (T = 1, placebo = 0), two binary indicators denoting description as power-enhancing or status-enhancing (i.e., quality was the baseline), and interaction terms between treatment and the two description conditions (i.e., power and status). A positive interaction implies that T-treated participants put a greater weight on the corresponding attribute compared to the placebo group; the binary treatment effect estimates the weight that the T-treated participants put on the baseline "quality" description compared to the placebo group.

All analyses were conducted using the function *lmer* implemented in R.[14] Model B1 included all the IVs discussed above; model B2 also included age, positive and negative affect, treatment expectancy, and the right hand 2D:4D (results hold when the left hand or the average between the two hands is used). Model B3 included all of the IVs of model B2 and all of the hormonal levels that were not affected by the treatment, as measured from the first post-treatment saliva sample. We replicated model B3's results when the measurements were replaced by either the second post-treatment saliva sample (i.e., the third overall measurement, model B4) or the baseline (morning) saliva sample (model B5). The results are summarized in Supplementary Table 8.

We further tested the treatment's influence for each description condition in isolation, using a simple slope analysis estimating model B1 separately for quality (model C1), power (model C2), and status (model C3) ads (Supplementary Table 9).

Finally, we directly tested for differences in attitudes toward power and status ads by applying the same analytical strategy to the power and status ads alone (i.e., excluding quality), when employing the power category as the baseline (Supplementary Table 11).

Testing for a dispositional effect of T on attitudes toward goods promoting different strategies to enhance social rank. We repeated the analyses above (testing for the influence of

T administration on task 2), this time when including either the logged baseline (pre-treatment) T levels, or the baseline levels of androstenedione.

Model G1, G2, and G3 are identical to models B1, B2, and B3, but also include the logged baseline T levels. Models G4, G5, and G6 repeated these analyses, this time with baseline levels of androstenedione instead of testosterone. The results are summarized in Supplementary Table 13.

T's influence on hypothetical purchase behavior for goods promoting different strategies to enhance social rank. We applied the same analytic strategy we used for the attitude scores to investigate the influence of T on the secondary measure of general purchase intentions. The models also included controls for ownership status and general buying intentions for the good category (self-reported in the post-experimental survey); both were significant predictors ($p < 0.01$) of the DV. The results are summarized in Supplementary Table 10.

Supplementary Figure 1. Summary of pretest of text descriptions: Mean quality, power and status perception ratings of the text descriptions across the three conditions focusing on (1) quality, (2) power, and (3) status. Error bars denote s.e.m.

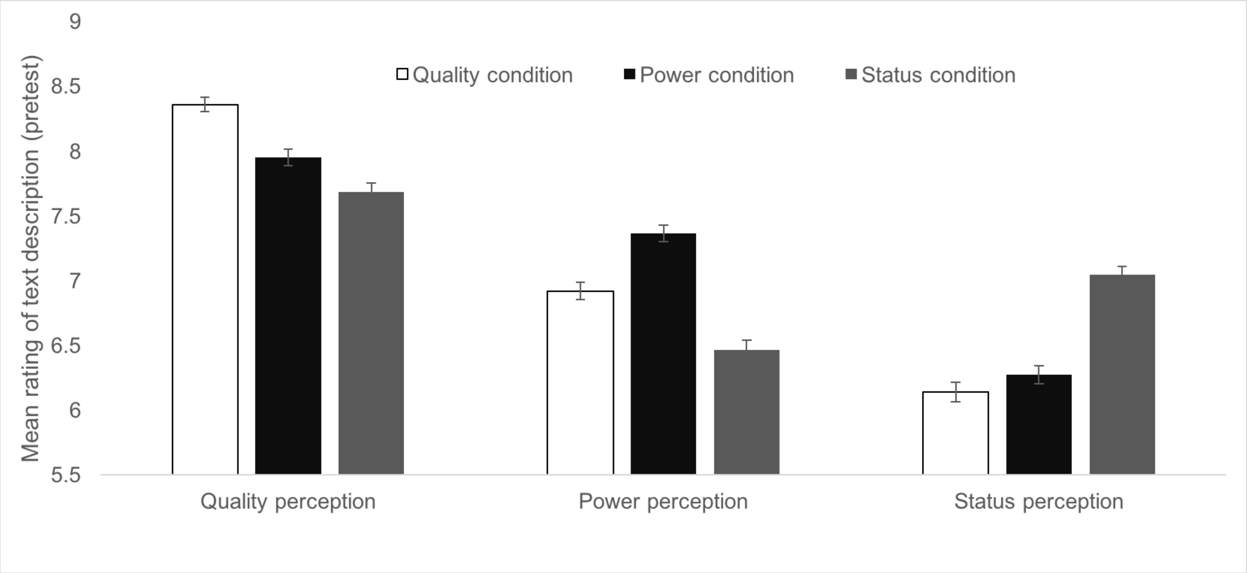

Supplementary Figure 2. Preference toward the high (versus low) social rank brands for the two treatment groups (z-scored at the pair level).

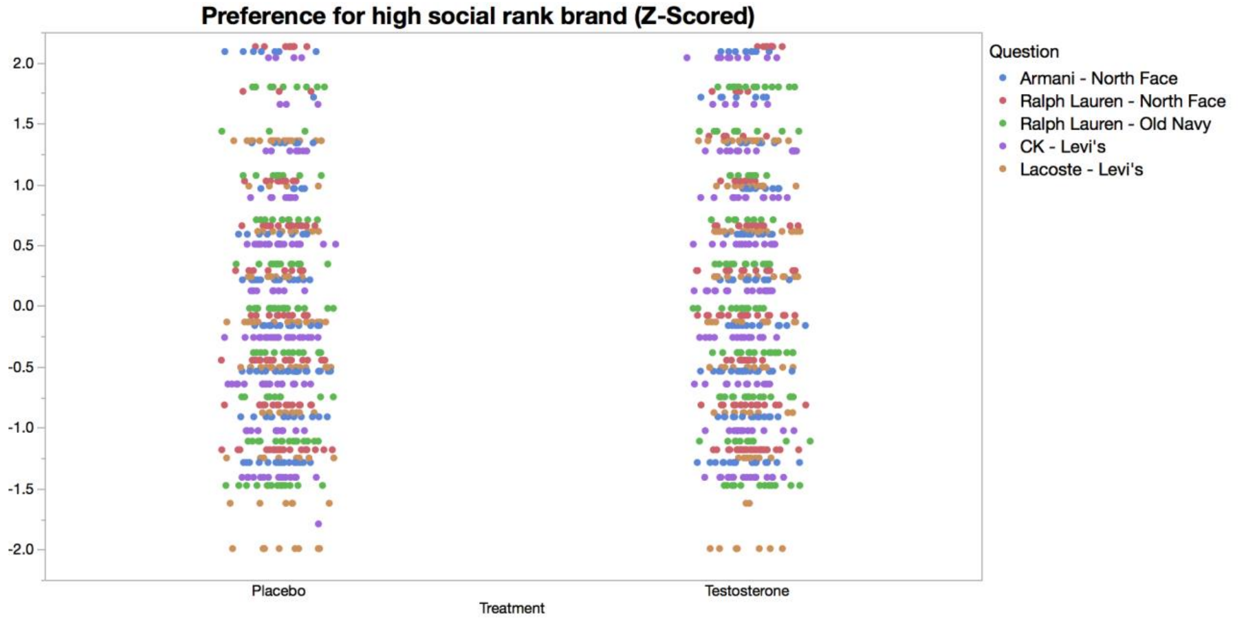

Supplementary Figure 3. Attitudes towards goods positioned as either status (Status = 1) or non-status (i.e., quality, power, Status=0) by treatment condition (z-scored at the ad level).

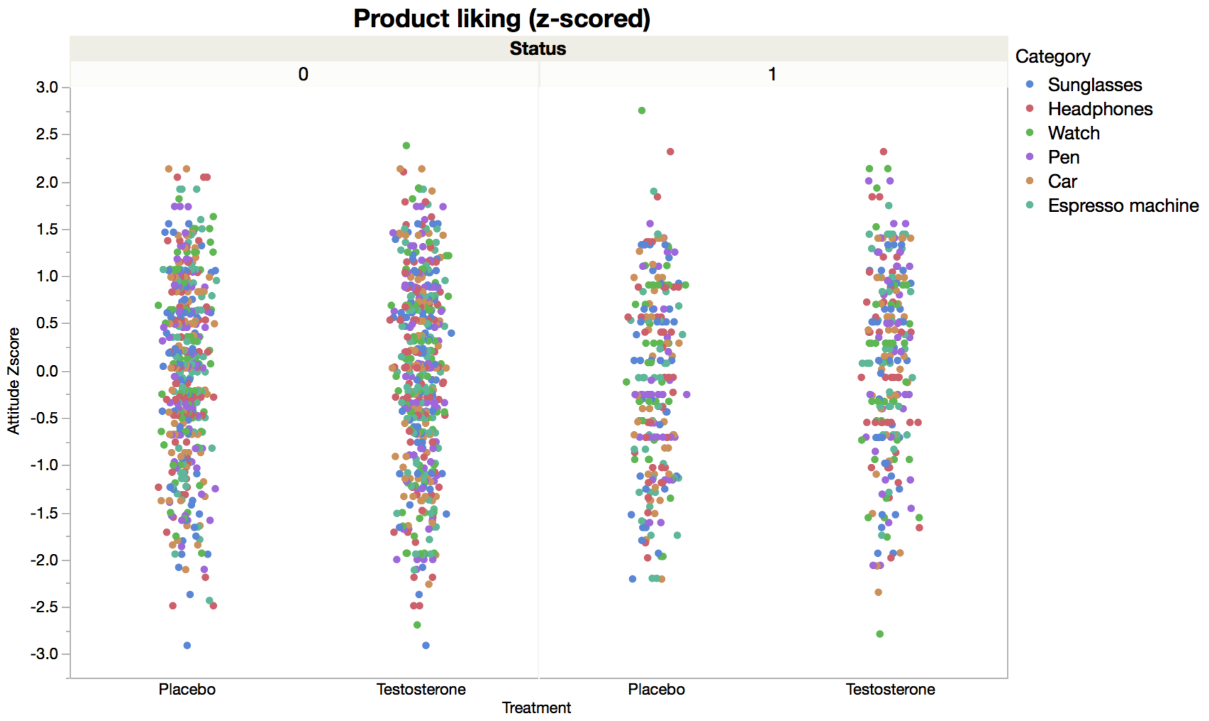

Supplementary Table 1. Self-reported demographics (standard errors in parentheses)

	All	T	Placebo	p-values for t-test of difference
**N**	243	118	125	
**Age**	23.63<br>(0.46)	24.42<br>(0.77)	22.78<br>(0.49)	0.08
**Left-handed (proportion)**	0.074<br>(0.02)	0.064<br>(0.02)	0.085<br>(0.03)	0.54
**Heterosexual (proportion)**	0.90<br>(0.02)	0.91<br>(0.03)	0.89<br>(0.03)	0.56
**Treatment expectancy[1]**	2.76<br>(0.06)	2.67<br>(0.08)	2.85<br>(0.09)	0.16
**Married (proportion)**	0.08<br>(0.02)	0.09<br>(0.03)	0.08<br>(0.03)	0.74
**In a relationship (proportion)**	0.38<br>(0.03)	0.34<br>(0.05)	0.42<br>(0.04)	0.20
**Has children**	0.06<br>(0.02)	0.08<br>(0.02)	0.04<br>(0.02)	0.23
**Personal monthly income[2]**	2.05<br>(0.11)	2.02<br>(0.14)	2.07<br>(0.16)	0.84

[1] 5-point scale: 1 = definitely did not get T, 2 = probably not, 3 = unsure, 4 = probably got T, 5 = definitely got T

[2] 5-point scale: 1 = under $500/month, 2 = $501–$1,000/month, 3 = $1,001–$1,500/month, 4 = $1,501–$2,000/month, 5 = over $2001/month

Supplementary Table 2. Detection levels, precision, and normality tests of hormonal assay

Analyte (pg/ml)	LLOQ	Range	Precision	Proportion undetected, sample A	Intra-assay COV	Inter-assay COV	Proportion undetected, sample B	K-S test p-value	K-S test (log) p-value
**Estrone**	0.5	0.5–510	8.7–13.7%	0.132	7.9%	11.9%	0.257	<0.01	0.56
**Estradiol**	0.3	0.3–510	4.3–18.7%	0.128	8.7%	12.2%	0.329	0.06	0.88
**Testosterone**	3.0	3.0–5100	3.0–18.1%	0	4.7%	10.3%	0.008	$<10^{-20}$	<0.01
**Androstenedione**	5.0	5.0–2300	5.2–6.6%	0	5.3%	5.3%	0.008	$<10^{-20}$	0.008
**DHEA**	20.0	20.0–1800	4.1–15.2%	0.004	6.7%	10.5%	0.012	0.002	0.98
**DHT**	10.0	10.0–920	3.6–17.7%	0.786	6.6%	14.8%	0.473	$<10^{-11}$	0.02
**Progesterone**	10.0	10.0–10000	4.8–10.8%	0.794	5.1%	9.5%	0.753	<0.01	0.03
**17OH-Progesterone**	5.0	5.0–630	3.9–13.8%	0.004	6.1%	11.1%	0.061	0.003	0.98
**11-Deoxycortisol**	5.0	5.0–410	6.8–16.6%	0.132	6.3%	9.2%	0.473	<0.01	0.04
**Cortisol**	0.1	0.1–52	5.1–17.9%	0	4.5%	10.7%	0.008	<0.01	0.92
**Cortisone**	0.1	0.1–81	4.1–14.9%	0	5.6%	8.2%	0.008	0.07	0.59
**Corticosterone**	5.0	5.0–1800	4.6–17.5%	0.313	7.9%	10.0%	0.312	<0.01	0.08
**Aldosterone**	10.0	10.0–560	8.9–18.8%	0.272	6.2%	14.9%	0.272	<0.06	0.39
**Melatonin**	2.5	2.5–10000	5.2–15.9%	0.502	6.9%	9.1%	0.500	0.07	0.14

Note: p-values are calculated using a Kolmogorov-Smirnov test for the distributions of the second saliva sample compared to Gaussian, and for the log-transform (the null hypothesis is normal Gaussian distribution).

Supplementary Table 3. Hormonal measurements in log(pg/mL) (standard errors in parentheses)

	**Placebo**		**Testosterone**		**Two-tailed p-value from t-test of T–placebo equality**	
**Sampling time**[1]	9 a.m.	2 p.m.	9 a.m.	2 p.m.	9 a.m.	2 p.m.
**Testosterone**	5.743	5.111	5.580	8.373	0.267	1.06E-13
	(0.085)	(0.085)	(0.084)	(0.151)		
**Androstenedione**	4.510	4.205	4.525	5.462	0.634	3.11E-09
	(0.039)	(0.044)	(0.034)	(0.084)		
**DHT**	1.984	1.867	1.905	3.482	0.745	2.38E-06
	(0.069)	(0.051)	(0.060)	(0.114)		
**Progesterone**	1.937	2.002	1.829	1.883	0.36	0.41
	(0.058)	(0.064)	(0.052)	(0.055)		
**Progesterone17OH**	3.245	2.675	3.217	2.463	0.792	0.008
	(0.050)	(0.058)	(0.049)	(0.058)		
**Estrone**	–0.088	–0.557	–0.007	–0.389	0.29	0.42
	(0.063)	(0.066)	(0.064)	(0.056)		
**Estradiol**	–0.743	–1.158	–0.766	–1.066	0.86	0.44
	(0.052)	(0.059)	(0.054)	(0.054)		
**DHEA**	5.198	4.570	5.116	4.557	0.30	0.76
	(0.053)	(0.058)	(0.051)	(0.054)		
**Deoxycortisol11**	2.579	1.650	2.568	1.584	0.66	0.35
	(0.079)	(0.072)	(0.083)	(0.064)		
**Cortisol**	1.047	0.062	1.045	0.077	0.68	0.81

	(0.058)	(0.065)	(0.057)	(0.058)		
**Cortisone**	2.539	1.952	2.539	2.003	0.70	0.76
	(0.030)	(0.060)	(0.034)	(0.050)		
**Corticosterone**	2.442	1.274	2.646	1.290	0.37	0.76
	(0.126)	(0.065)	(0.123)	(0.060)		
**Aldosterone**	2.640	2.516	2.634	2.395	0.82	0.14
	(0.067)	(0.071)	(0.068)	(0.066)		
**Melatonin**	1.045	0.276	1.221	0.353	0.27	0.23
	(0.093)	(0.029)	(0.101)	(0.051)		

[1] Main effects of time (afternoon vs. treatment) are due to the diurnal cycles of the hormones. [5-7]

Supplementary Table 4. Positive/negative affect (PANAS) summary statistics

	All		Testosterone		Placebo		ANOVA: p-values		
**Time**	**Morning**	**Afternoon**	**Morning**	**Afternoon**	**Morning**	**Afternoon**	**T**	**Time**	**T x time**
**Positive affect**	2.72 (0.05)	2.61 (0.06)	2.72 (0.06)	2.63 (0.08)	2.72 (0.07)	2.60 (0.09)	0.85	0.16	0.85
**Negative affect**	1.53 (0.04)	1.45 (0.04)	1.53 (0.06)	1.46 (0.05)	1.53 (0.05)	1.43 (0.05)	0.77	0.13	0.84

Supplementary Table 5. Manipulation check of brand associations. The table summarizes the participants' quality and social rank (combination of power and status) associations of the five pair brands used in the experimental task. The two right columns denote the 95% confidence interval of the difference in difference between the brands' associations with social rank and quality.

Brand H	Brand L	Rank H	Rank L	Rank Diff.	Quality H	Quality L	Quality Diff.	Diff. in diff. 95% CI
**Giorgio Armani**	**North Face**	80.15	46.39	33.76	73.48	77.75	–4.27	[33.33 42.73]
**Ralph Lauren**	**North Face**	68.25	46.39	21.86	67.21	77.75	–10.45	[27.89 36.72]
**Ralph Lauren**	**Old Navy**	68.25	20.20	47.95	67.21	36.88	30.32	[12.87 22.40]
**Calvin Klein**	**Levi's**	60.61	35.83	24.75	64.09	73.67	–9.58	[29.79 38.88]
**Lacoste**	**Levi's**	57.57	35.83	21.78	58.81	73.67	–15.15	[31.73 42.12]

Supplementary Table 6. T's influence on brand associations. Mean quality and social rank associations of the different brands across the two treatment groups, and 95% confidence interval of a two-sample t-test comparing them.

	Quality			Social rank		
	**T**	**Placebo**	**95% CI of Diff**	**T**	**Placebo**	**95% CI of Diff**
**Giorgio Armani**	60.08	57.43	[–3.690 7.391]	56.30	58.93	[–3.075 6.385]
**Ralph Lauren**	68.86	65.47	[–1.935 8.711]	68.00	68.51	[–5.845 4.836]
**Calvin Klein**	67.70	60.23	[1.849 13.098]*	60.97	60.24	[–5.090 6.559]
**Lacoste**	60.08	57.43	[–3.723 9.025]	56.30	58.93	[–9.188 3.930]
**North Face**	75.80	79.81	[–9.158 1.131]	44.77	48.09	[–8.996 2.342]
**Old Navy**	36.56	37.21	[–6.663 5.348]	20.27	20.13	[–4.594 4.861]
**Levi's**	73.82	73.52	[–5.050 5.639]	36.47	35.16	[–4.180 6.799]

Note: $^{*}p < 0.01$

Supplementary Table 7: T's influence on preference for brands high in social rank (task 1), estimated using mixed model linear regressions with participant and brand random intercepts. Hormonal measurements are logged; standard errors in parentheses.

	**Dependent variable: Brand preference rating (z-scored)**				
	**(A1)**	**(A2)**	**(A3)**	**(A4)**	**(A5)**
Treatment	0.182**	0.215**	0.190*	0.173*	0.232**
	(0.091)	(0.096)	(0.100)	(0.100)	(0.097)
Age		–0.015**	–0.012*	–0.017**	–0.010
		(0.007)	(0.007)	(0.007)	(0.007)
Negative affect		–0.060	–0.062	–0.100	–0.099
		(0.089)	(0.094)	(0.092)	(0.090)
Positive affect		0.017	0.027	0.024	0.030
		(0.050)	(0.052)	(0.052)	(0.051)
Belief		0.076	0.092*	0.087*	0.097*
		(0.051)	(0.052)	(0.052)	(0.051)
Digit ratio (right)		1.137	0.903	1.459	1.463
		(1.368)	(1.402)	(1.407)	(1.368)
Estrone			0.026	0.067	–0.172**
			(0.078)	(0.074)	(0.081)
Estradiol			–0.010	0.048	0.138
			(0.082)	(0.083)	(0.092)
DHEA			0.049	0.069	0.105
			(0.103)	(0.089)	(0.100)
Progesterone			–0.001	–0.139	–0.130
			(0.074)	(0.086)	(0.082)
Deoxycortisol11			0.037	–0.030	0.096
			(0.091)	(0.085)	(0.086)
Cortisol			–0.205	0.012	–0.180
			(0.156)	(0.113)	(0.159)
Cortisone			0.352*	0.165	0.153
			(0.205)	(0.122)	(0.257)
Corticosterone			0.043	–0.144	–0.066
			(0.082)	(0.099)	(0.057)
Aldosterone			0.049	0.111	0.158**
			(0.066)	(0.101)	(0.068)
Melatonin			0.019	0.088	0.048
			(0.106)	(0.112)	(0.052)
Constant	–0.094	–1.020	–2.066	–1.562	–2.426
	(0.065)	(1.317)	(1.476)	(1.477)	(1.504)
Observations	1,215	1,145	1,135	1,145	1,145
Log likelihood	–1,603.223	–1,513.236	–1,511.742	–1,522.513	–1,521.555
Akaike inf. crit.	3,216.446	3,046.472	3,063.485	3,085.025	3,083.110
Bayesian inf. crit.	3,241.959	3,096.903	3,164.172	3,185.888	3,183.973
Note:	$^{*}p < 0.1$ $^{**}p < 0.05$ $^{***}p < 0.01$				

Supplementary Table 8. Treatment influence on attitudes toward goods (task 2), estimated using mixed model linear regressions with participant random intercepts and slopes for "power" and "status" positioning, and product random intercepts. Hormonal measurements are logged; standard errors in parentheses.

	**Dependent variable:**				
	**Attitude score (z-scored)**				
	**(B1)**	**(B2)**	**(B3)**	**(B4)**	**(B5)**
Treatment	-0.044	-0.086	-0.044	-0.044	-0.048
	(0.102)	(0.104)	(0.105)	(0.105)	(0.106)
Power	0.007	0.011	0.029	0.029	0.011
	(0.082)	(0.085)	(0.084)	(0.084)	(0.085)
Treatment x Power	-0.017	-0.027	-0.031	-0.031	-0.027
	(0.115)	(0.119)	(0.118)	(0.118)	(0.119)
Status	-0.143*	-0.152*	-0.133	-0.133	-0.152*
	(0.085)	(0.086)	(0.086)	(0.086)	(0.086)
Treatment x Status	0.275**	0.249**	0.237**	0.237**	0.249**
	(0.119)	(0.120)	(0.120)	(0.120)	(0.120)
Age		0.011**	0.014**	0.014**	0.012**
		(0.005)	(0.006)	(0.006)	(0.006)
Negative affect		0.112	0.136*	0.136*	0.099
		(0.070)	(0.074)	(0.074)	(0.073)
Positive affect		0.113***	0.103**	0.103**	0.114***
		(0.040)	(0.041)	(0.041)	(0.041)
Belief		-0.050	-0.043	-0.043	-0.045
		(0.041)	(0.041)	(0.041)	(0.042)
Digit ratio (right)		-0.855	-1.022	-1.022	-0.736
		(1.085)	(1.107)	(1.107)	(1.121)
Estrone			-0.055	-0.055	-0.101*
			(0.061)	(0.061)	(0.059)
Estradiol			-0.139**	-0.139**	-0.057
			(0.065)	(0.065)	(0.066)
DHEA			0.037	0.037	-0.006
			(0.081)	(0.081)	(0.071)
Progesterone			0.052	0.052	-0.077
			(0.059)	(0.059)	(0.068)
Deoxycortisol11			-0.010	-0.010	0.027
			(0.072)	(0.072)	(0.068)
Cortisol			-0.164	-0.164	-0.008
			(0.123)	(0.123)	(0.090)

Cortisone			0.057	0.057	-0.072
			(0.162)	(0.162)	(0.097)
Corticosterone			0.061	0.061	-0.038
			(0.065)	(0.065)	(0.079)
Aldosterone			0.053	0.053	0.078
			(0.052)	(0.052)	(0.080)
Melatonin			0.017	0.017	0.010
			(0.083)	(0.083)	(0.089)
Constant	0.024	0.314	-0.405	-0.405	0.200
	(0.073)	(1.046)	(1.166)	(1.166)	(1.177)
Observations	1,446	1,362	1,350	1,350	1,362
Log Likelihood	-1,995.502	-1,867.973	-1,860.620	-1,860.620	-1,880.624
Akaike Inf. Crit.	4,019.004	3,773.945	3,779.241	3,779.241	3,819.248
Bayesian Inf. Crit.	4,092.875	3,873.063	3,930.269	3,930.269	3,970.533
Note:					$^{*}p < 0.1$ $^{**}p < 0.05$ $^{***}p < 0.01$

Supplementary Table 9. Treatment influence on attitude toward the goods, by positioning condition). Each column shows the results of a linear regression analysis using a mixed model with participant and product random intercepts.

	**Dependent variable:**		
	**Attitude score (z-scored)**		
	**(C1) Quality**	**(C2) Power**	**(C3) Status**
Treatment	–0.046	–0.063	0.231**
	(0.102)	(0.102)	(0.099)
Constant	0.025	0.031	–0.119*
	(0.073)	(0.073)	(0.071)
Observations	482	481	483
Log likelihood	–676.661	–673.603	–677.509
Akaike inf. crit.	1,363.322	1,357.205	1,365.018
Bayesian inf. crit.	1,384.211	1,378.085	1,385.918
Note:	*$p < 0.1$ **$p < 0.05$ ***$p < 0.01$		

Supplementary Table 10. Treatment influence on hypothetical purchase behavior for goods (task 2), estimated using mixed model linear regressions with participant random intercepts and slopes for "power" and "status" positioning, and product random intercepts. Hormonal measurements are logged, standard errors in parentheses.

	**Dependent variable:** **General purchase intentions**				
	**(D1)**	**(D2)**	**(D3)**	**(D4)**	**(D5)**
Treatment	-0.042	-0.070	-0.063	-0.048	-0.076
	(0.084)	(0.085)	(0.088)	(0.089)	(0.087)
Power	0.044	0.065	0.070	0.065	0.066
	(0.072)	(0.073)	(0.073)	(0.073)	(0.073)
Treatment x Power	0.020	0.011	0.014	0.010	0.010
	(0.099)	(0.101)	(0.102)	(0.101)	(0.102)
Status	-0.109	-0.090	-0.079	-0.089	-0.089
	(0.067)	(0.069)	(0.069)	(0.069)	(0.069)
Treatment x Status	0.160*	0.138	0.135	0.138	0.138
	(0.093)	(0.096)	(0.096)	(0.096)	(0.096)
Buy	0.057***	0.058***	0.057***	0.058***	0.057***
	(0.010)	(0.011)	(0.011)	(0.011)	(0.011)
Own	0.139***	0.146***	0.136***	0.148***	0.149***
	(0.049)	(0.051)	(0.051)	(0.051)	(0.051)
Age		0.008*	0.009*	0.010*	0.008
		(0.005)	(0.005)	(0.005)	(0.005)
Negative affect		0.073	0.089	0.068	0.084
		(0.063)	(0.067)	(0.066)	(0.065)
Positive affect		0.103***	0.099**	0.114***	0.110***
		(0.037)	(0.038)	(0.039)	(0.038)
Belief		-0.072*	-0.074*	-0.067*	-0.071*
		(0.037)	(0.038)	(0.038)	(0.038)
Digit Ratio (right)		-0.687	-0.818	-0.556	-0.644
		(0.981)	(1.014)	(1.019)	(0.996)
Estrone			0.009	-0.035	0.010
			(0.055)	(0.053)	(0.058)
Estradiol			-0.009	-0.016	0.012
			(0.058)	(0.059)	(0.065)
DHEA			0.065	0.070	0.096
			(0.073)	(0.064)	(0.072)
Progesterone			0.012	-0.046	-0.053
			(0.053)	(0.061)	(0.060)
Deoxycortisol11			0.061	0.038	0.078

			(0.065)	(0.061)	(0.062)
Cortisol			-0.246**	-0.126	-0.159
			(0.111)	(0.081)	(0.114)
Cortisone			0.123	-0.053	-0.164
			(0.146)	(0.087)	(0.184)
Corticosterone			0.070	0.033	0.039
			(0.059)	(0.071)	(0.041)
Aldosterone			0.009	0.047	0.018
			(0.048)	(0.072)	(0.049)
Melatonin			-0.035	0.030	-0.030
			(0.075)	(0.079)	(0.037)
				-0.035	0.010
Constant	-0.211***	0.076	-0.569	-0.510	-0.109
	(0.076)	(0.945)	(1.064)	(1.065)	(1.087)
Observations	1,401	1,330	1,318	1,330	1,330
Log Likelihood	-1,678.013	-1,595.558	-1,591.104	-1,610.562	-1,609.879
Akaike Inf. Crit.	3,388.026	3,233.117	3,244.208	3,283.123	3,281.757
Bayesian Inf. Crit.	3,471.945	3,342.169	3,404.908	3,444.104	3,442.738
Note:					*$p < 0.1$ **$p < 0.05$ ***$p < 0.01$

Supplementary Table 11. Treatment influence on attitudes toward goods (task 2), mixed model linear regression with participant random intercepts and slopes for "power" and "status" positioning, and product random intercepts. The data includes only the "power" and "status" conditions, with "power" as the baseline. Hormonal measurements are logged; standard errors in parentheses.

	**Dependent variable: Attitude score (z-scored)**				
	**(E1)**	**(E2)**	**(E3)**	**(E4)**	**(E5)**
Treatment	-0.061	-0.112	-0.061	-0.085	-0.091
	(0.102)	(0.105)	(0.106)	(0.108)	(0.106)
Status	-0.150*	-0.164*	-0.162*	-0.164*	-0.164*
	(0.086)	(0.088)	(0.088)	(0.088)	(0.088)
Treatment x Status	0.293**	0.278**	0.270**	0.278**	0.278**
	(0.121)	(0.122)	(0.123)	(0.122)	(0.122)
Age		0.010*	0.014**	0.012*	0.011*
		(0.006)	(0.006)	(0.006)	(0.006)
Negative affect		0.141*	0.151*	0.130	0.153*
		(0.077)	(0.081)	(0.080)	(0.080)
Positive affect		0.114***	0.099**	0.114**	0.102**
		(0.044)	(0.045)	(0.045)	(0.045)
Belief		-0.066	-0.054	-0.061	-0.065
		(0.044)	(0.045)	(0.046)	(0.045)
Digit Ratio (right)		-0.403	-0.674	-0.297	-0.191
		(1.185)	(1.204)	(1.230)	(1.205)
Estrone			-0.055	-0.094	-0.060
			(0.067)	(0.065)	(0.071)
Estradiol			-0.159**	-0.083	-0.089
			(0.070)	(0.073)	(0.081)
DHEA			0.073	0.024	0.109
			(0.088)	(0.078)	(0.088)
Progesterone			0.100	-0.066	-0.029
			(0.064)	(0.075)	(0.073)
Deoxycortisol11			-0.001	-0.031	0.078
			(0.079)	(0.075)	(0.076)
Cortisol			-0.125	0.027	0.071
			(0.134)	(0.099)	(0.140)
Cortisone			0.024	-0.018	-0.180
			(0.176)	(0.107)	(0.229)
Corticosterone			0.028	-0.051	-0.035

			(0.071)	(0.087)	(0.050)
Aldosterone			0.082	0.110	-0.034
			(0.057)	(0.088)	(0.060)
Melatonin			0.014	-0.006	-0.028
			(0.091)	(0.098)	(0.045)
Constant	0.031	-0.084	-0.955	-0.414	-0.513
	(0.073)	(1.142)	(1.270)	(1.292)	(1.327)
Observations	964	908	900	908	908
Log Likelihood	-1,341.313	-1,257.150	-1,256.238	-1,269.867	-1,270.497
Akaike Inf. Crit.	2,700.627	2,542.300	2,560.477	2,587.734	2,588.995
Bayesian Inf. Crit.	2,744.467	2,609.657	2,675.734	2,703.203	2,704.464
Note:					$^{*}p < 0.1$ $^{**}p < 0.05$ $^{***}p < 0.01$

Supplementary Table 12: The influence of baseline testosterone and androstenedione levels on preference for brands high in social rank (task 1), mixed model linear regression with participant and brand random intercepts. Hormonal measurements are logged; standard errors in parentheses.

	**Dependent variable: Brand preference rating (z-scored)**					
	**(F1)**	**(F2)**	**(F3)**	**(F4)**	**(F5)**	**(F6)**
Testosterone (baseline)	0.130***	0.127**	0.138***			
	(0.046)	(0.050)	(0.052)			
Androstenedione (baseline)				0.301***	0.251**	0.205
				(0.111)	(0.119)	(0.131)
Treatment	0.206**	0.223**	0.197**	0.182**	0.207**	0.185*
	(0.090)	(0.095)	(0.098)	(0.089)	(0.095)	(0.099)
Age		–0.012*	–0.010		–0.011*	–0.010
		(0.006)	(0.007)		(0.007)	(0.007)
Negative affect		–0.061	–0.070		–0.055	–0.058
		(0.088)	(0.092)		(0.088)	(0.093)
Positive affect		0.033	0.047		0.028	0.036
		(0.050)	(0.052)		(0.050)	(0.052)
Belief		0.073	0.090*		0.074	0.087*
		(0.051)	(0.052)		(0.051)	(0.052)
Digit ratio (right)		0.808	0.658		1.091	0.912
		(1.358)	(1.385)		(1.358)	(1.397)
Estrone			0.025			0.026
			(0.076)			(0.077)
Estradiol			0.020			–0.009
			(0.081)			(0.082)
DHEA			0.040			0.016
			(0.101)			(0.104)
Progesterone			–0.014			–0.005
			(0.073)			(0.074)
Deoxycortisol11			0.029			0.029
			(0.090)			(0.091)
Cortisol			–0.205			–0.168
			(0.154)			(0.157)
Cortisone			0.360*			0.283
			(0.202)			(0.209)
Corticosterone			0.019			0.045
			(0.082)			(0.082)
Aldosterone			0.050			0.034

			(0.065)			(0.067)
Melatonin			0.026			0.011
			(0.104)			(0.105)
Constant	–0.846***	–1.512	–2.563*	–1.456***	–2.217	–2.719*
	(0.270)	(1.315)	(1.466)	(0.507)	(1.425)	(1.529)
Observations	1,215	1,145	1,135	1,215	1,145	1,135
Log likelihood	–1,601.338	–1,512.110	–1,510.239	–1,600.875	–1,512.243	–1,511.638
Akaike inf. crit.	3,214.676	3,046.220	3,062.477	3,213.749	3,046.486	3,065.276
Bayesian inf. crit.	3,245.291	3,101.695	3,168.199	3,244.364	3,101.961	3,170.998
Note:	$^{*}p < 0.1$ $^{**}p < 0.05$ $^{***}p < 0.01$					

Supplementary Table 13. The influence of baseline testosterone and androstenedione levels on attitudes toward goods (task 2), mixed model linear regression with participant random intercepts and slopes for "power" and "status" positioning, and product random intercepts. Hormonal measurements are logged; standard errors in parentheses.

	**Dependent variable: Attitude score (z-scored)**					
	**(G1)**	**(G2)**	**(G3)**	**(G4)**	**(G5)**	**(G6)**
Testosterone (baseline)	-0.029	-0.010	-0.025			
	(0.052)	(0.055)	(0.056)			
Testosterone × Power	0.045	0.042	0.062			
	(0.059)	(0.063)	(0.063)			
Testosterone × Status	0.039	0.050	0.062			
	(0.061)	(0.064)	(0.064)			
Androstenedione (baseline)				-0.020	0.046	0.041
				(0.129)	(0.132)	(0.138)
Androstenedione × Power				0.018	0.081	0.122
				(0.147)	(0.150)	(0.149)
Androstenedione × Status				-0.063	-0.023	0.009
				(0.152)	(0.153)	(0.152)
Treatment	-0.049	-0.088	-0.048	-0.043	-0.088	-0.045
	(0.102)	(0.104)	(0.105)	(0.102)	(0.104)	(0.105)
Treatment × Power	-0.009	-0.023	-0.023	-0.017	-0.029	-0.033
	(0.116)	(0.119)	(0.118)	(0.115)	(0.119)	(0.118)
Treatment × Status	0.282**	0.255**	0.245**	0.276**	0.250**	0.237**
	(0.120)	(0.121)	(0.120)	(0.119)	(0.121)	(0.120)
Power	-0.254	-0.231	-0.327	-0.073	-0.354	-0.521
	(0.350)	(0.371)	(0.369)	(0.668)	(0.685)	(0.679)
Status	-0.367	-0.439	-0.490	0.140	-0.050	-0.174
	(0.362)	(0.375)	(0.375)	(0.691)	(0.695)	(0.691)
Age		0.011**	0.014**		0.011**	0.014**
		(0.005)	(0.006)		(0.005)	(0.006)
Negative affect		0.113	0.135*		0.114	0.138*
		(0.070)	(0.074)		(0.070)	(0.074)
Positive affect		0.116***	0.106**		0.116***	0.108***
		(0.040)	(0.041)		(0.040)	(0.041)
Belief		-0.051	-0.043		-0.050	-0.044
		(0.041)	(0.041)		(0.041)	(0.041)
Digit Ratio (right)		-0.912	-1.054		-0.873	-1.030
		(1.092)	(1.112)		(1.086)	(1.108)
Estrone			-0.055			-0.056
			(0.061)			(0.061)

Estradiol			-0.135^{**}			-0.139^{**}
			(0.065)			(0.065)
DHEA			0.036			0.024
			(0.081)			(0.083)
Progesterone			0.051			0.051
			(0.059)			(0.059)
Deoxycortisol11			-0.011			-0.014
			(0.072)			(0.073)
Cortisol			-0.163			-0.148
			(0.123)			(0.125)
Cortisone			0.058			0.030
			(0.162)			(0.166)
Corticosterone			0.057			0.060
			(0.066)			(0.065)
Aldosterone			0.053			0.046
			(0.053)			(0.053)
Melatonin			0.018			0.014
			(0.084)			(0.083)
Constant	0.193	0.412	-0.223	0.117	0.099	-0.469
	(0.309)	(1.080)	(1.197)	(0.590)	(1.211)	(1.278)
Observations	1,446	1,362	1,350	1,446	1,362	1,350
Log Likelihood	-2,001.427	-1,873.562	-1,865.931	-1,998.807	-1,870.991	-1,863.298
Akaike Inf. Crit.	4,040.854	3,791.124	3,795.863	4,035.613	3,785.982	3,790.596
Bayesian Inf. Crit.	4,141.109	3,905.891	3,962.514	4,135.868	3,900.749	3,957.248
Note:						$^{*}p < 0.1$ $^{**}p < 0.05$ $^{***}p < 0.01$

Supplementary References

1. Ronay, R. & von Hippel, W. The presence of an attractive woman elevates testosterone and physical risk taking in young men. *Soc. Psychol. Personal. Sci.* **1,** 57–64 (2010).

2. Horton, R. & Tait, J. F. Androstenedione production and interconversion rates measured in peripheral blood and studies on the possible site of its conversion to testosterone. *J. Clin. Invest.* **45,** 301–313 (1966).

3. Mayo, A., Macintyre, H., Wallace, A. M. & Ahmed, S. F. Transdermal testosterone application: pharmacokinetics and effects on pubertal status, short-term growth, and bone turnover. *J. Clin. Endocrinol. Metab.* **89,** 681–687 (2004).

4. Du, J. Y., Sanchez, P., Kim, L., Azen, C. G., Zava, D. T. & Stanczyk, F. Z. Percutaneous progesterone delivery via cream or gel application in postmenopausal women: a randomized cross-over study of progesterone levels in serum, whole blood, saliva, and capillary blood. *Menopause* **20,** 1169–1175 (2013).

5. Nomura, S., Fujitaka, M., Sakura, N. & Ueda, K. Circadian rhythms in plasma cortisone and cortisol and the cortisone/cortisol ratio. *Clin. Chim. Acta.* **266,** 83–91 (1997).

6. Hucklebridge, F., Hussain, T., Evans, P. & Clow, A. The diurnal patterns of the adrenal steroids cortisol and dehydroepiandrosterone (DHEA) in relation to awakening. *Psychoneuroendocrino.* **30,** 51–57 (2005).

7. Hurwitz, S., Cohen, R. J. & Williams, G. H. Diurnal variation of aldosterone and plasma renin activity: timing relation to melatonin and cortisol and consistency after prolonged bed rest. *J. Appl. Physiol.* **96,** 1406–1414 (2004).

8. Rolf, C., Knie, U., Lemmnitz, G. & Nieschlag, E. Interpersonal testosterone transfer after topical application of a newly developed testosterone gel preparation. *Clin. Endocrinol.* **56,** 637–641 (2002).

9. Manning, J. T. *Digit Ratio: A Pointer to Fertility, Behavior, and Health* (Rutgers Series in Human Evolution, Rutgers University Press, New Brunswick, NJ, 2002).

10. Berenbaum, S. A., Bryk, K. K., Nowak, N., Quigley, C. A. & Moffat, S. Fingers as a marker of prenatal androgen exposure. *Endocrinology* **150,** 5119–5124 (2009).

11. Apicella, C. L., Tobolsky, V. A., Marlowe, F. W. & Miller, K. W. Hadza hunter-gatherer men do not have more masculine digit ratios (2D:4D). *Am. J. Phys. Anthropol.* **159,** 223–232 (2016).

12. Watson, D., Clark, L. A. & Tellegen, A. Development and validation of brief measures of positive and negative affect: the PANAS scales. *J. Pers. Soc. Psychol.* **54,** 1063–1070 (1988).

13. Barr, D. J., Levy, R., Scheepers, C. & Tily, H. J. Random effects structure for confirmatory hypothesis testing: keep it maximal. *J. Mem. Lang.* **68,** 255–278 (2013).

14. Bates, D. M. lme4: mixed-effects modeling with R (accessed July 19, 2017, at http://lme4.0.r-forge.r-project.org/lMMwR/lrgprt.pdf) (2010).

15. Tsutsui, K. & Ishii, S. Effects of sex steroids on aggressive behavior of adult male Japanese quail. *Gen. Comp. Endocr.* **44,** 480–486 (1981).

16. Payne, A. P. A comparison of the effects of androstenedione, dihydrotestosterone and testosterone propionate on aggression in the castrated male golden hamster. *Physiol. Behav.* **13.1**, 21–26 (1974).
